# Neural representation of the musical beat is facilitated but not contingent on the repetition of rhythmic patterns

**DOI:** 10.1038/s41598-025-30780-1

**Published:** 2025-12-17

**Authors:** Emmanuel Coulon, Sacha Baum, Tomas Lenc, Rainer Polak, Sylvie Nozaradan

**Affiliations:** 1 Institute of Neuroscience (IoNS), Université Catholique de Louvain (UCLouvain), Brussels, Belgium; 2 Basque Center On Cognition, Brain and Language, Donostia-San Sebastián, Spain; 3 RITMO Centre for Interdisciplinary Studies in Rhythm, Time and Motion, University of Oslo, Oslo, Norway; 4 International Laboratory for Brain, Music and Sound Research (BRAMS), Montreal, QC Canada

**Keywords:** Neuroscience, Psychology, Psychology

## Abstract

Music often entails perception of periodic beats which serve as internal temporal references to coordinate movements to music. Crucially, beat perception arises even in syncopated musical rhythms, which only weakly cue the beat periodicity. However, syncopated rhythms are often looped in music, suggesting that repetition of rhythmic patterns may facilitate beat perception by providing a periodic structure at a supra-second timescale. Here, we tested this hypothesis by recording separately electroencephalographic (EEG) and behavioral responses (finger tapping) while participants listened to different syncopated rhythmic sequences. These sequences either consisted of a repeated pattern (repetition of 4.8 and 9.6-s-long patterns) or were generated without repetition. Despite the degradation of pattern repetition, neural activity showed a periodized representation of the rhythmic input across conditions, at periodicities corresponding to those expressed in behavioral responses. However, this neural activity was further enhanced in the condition with shorter repeated patterns. Thus, pattern repetition was not necessary but strengthened the neural representation of the beat, demonstrating that supra-second periodicities in the rhythmic input further enhance sub-second periodicities in neural activity. These findings highlight the multiscale temporal processing of musical rhythm, and, more generally, complex rhythmic inputs involved in interpersonal interaction and communication.

**Supplementary Information:**

The online version contains supplementary material available at 10.1038/s41598-025-30780-1.

## Introduction

Music has a profound and universal effect on humans. This effect often results from movement (e.g., instrument playing, hand-clapping, singing, etc.), and also motivates movements (e.g., dancing, head bobbing, foot tapping, co-performers instrument playing, etc.) that are coordinated with the rhythmic input^1,2^. Coordinating rhythmic movements relies on an internal representation of time that is, to a great extent, invariant to the specific temporal arrangement of musical events. This internal representation is typically referred to as the beat, which consists of a seamlessly recurring pulse train elicited by a rhythmic input. Additionally to this particularly prominent beat, slower and faster pulse layers are often perceived, forming a multilayered structure of pulse trains called meter^3,4^.

Notably, beat representation can arise in response to a wide variety of rhythmic stimuli, ranging from controlled isochronous sequences (e.g., metronomes) to spectro-temporally complex real-world performances (e.g., marching bands, large orchestras, etc.). In other words, the brain forms a beat representation that does not necessarily correspond one-to-one to prominent features of rhythmic inputs. This phenomenon is illustrated well with syncopated rhythms, where the acoustic content only weakly cues the periodicity of the perceived beat. Yet, syncopated rhythms are commonly used in music and do not prevent listeners from perceiving a beat, as embodied in the production of periodic movement along with these rhythms^5–7^.

Analogously, studies using electrophysiology have repeatedly demonstrated that brain activity elicited while participants listen to syncopated rhythms shows a selective enhancement of periodicities corresponding to the perceived beat. In other words, there is robust evidence that beat perception is tied to processes that periodize the rhythmic sensory input, thus shaping the neural representation toward a behaviorally-relevant internal template^9–12^. Together, these findings highlight the fact that internal representations of the beat cannot be explained solely by sensory processing of prominent temporal cues of the input, but at least partially involve higher-level processes that enable a certain degree of invariance with respect to the input^13^.

Critically, meter perception concerns a specific temporal range lying between approximately 100 and 1800 ms (as delimited through ethnomusicological work, and behavioral experiments where participants were instructed to tap in synchrony along with isochronous rhythms^14–18^). It has thus been hypothesized that the temporal structure of the rhythmic input, covering this sub- to supra-second range, would provide temporal cues to map periodic pulses, making meter perception a multiscale scaffolding process, for example based on nonlinear neural responses to the stimulus^4,7,19–23^.

**Fig. 1:**
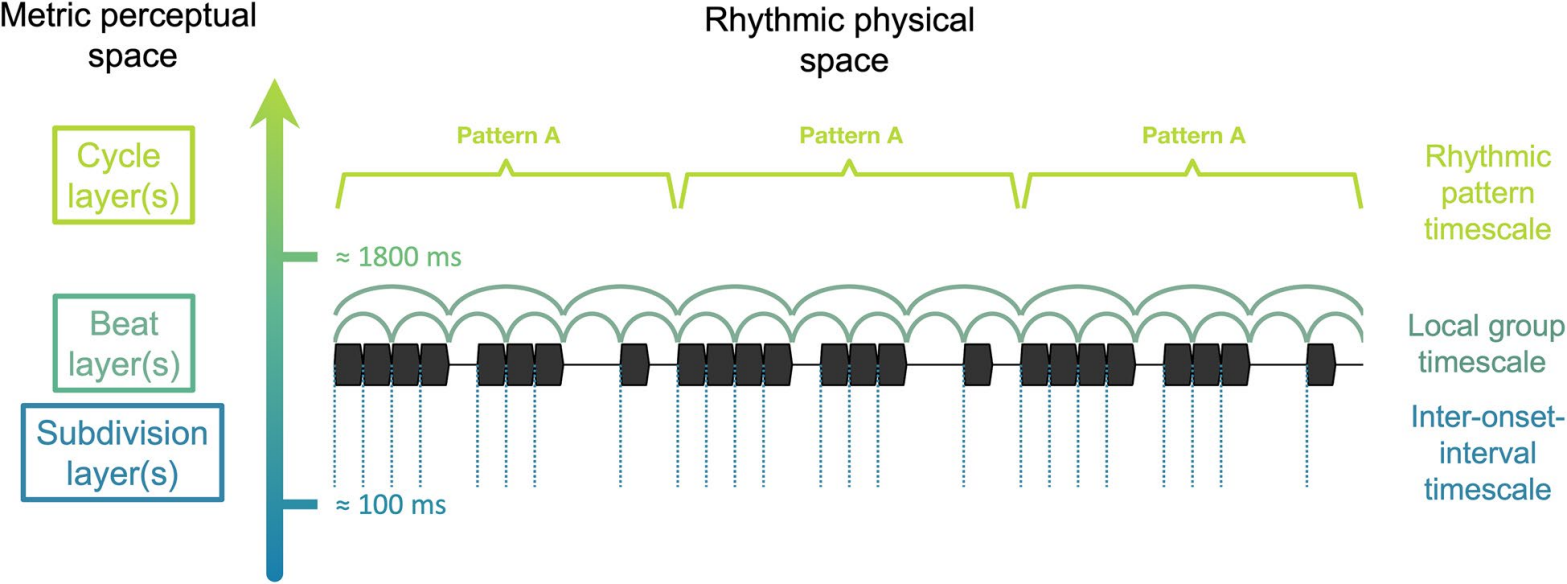
Three-layer scheme of the temporal range for meter and beat processing, showing the physical space of the rhythmic input and corresponding metric perceptual space. The figure depicts an example rhythmic sequence with prominent periodic cues in the faster layer, formed by recurrent intervals between successive sound events (dashed vertical lines) and prominent periodic cues in the slower layer, formed by the seamless repetition of the rhythmic pattern (as depicted with squared brackets). In contrast, the intermediate layer does not show prominent periodic cues due to the local arrangement of sound events forming a syncopated rhythm. Yet, most listeners can perceive at least one pulse layer in response to syncopated rhythms, as displayed by the nested half circles (depicting the pulse layers obtained in previous behavioral measurements^8^).

Here, we aimed to move a critical step forward in investigating this multiscale temporal scaffolding with a focus on its slower, supra-second end. We first propose a scheme where this temporal range for meter is organized into three pulse layers (Fig. 1). This scheme then serves as a basis to manipulate the prominence of temporal cues at the slow supra-second layer by varying the degree of pattern repetition, while keeping parameters at the faster sub-second timescale constant. This approach thus informs on the contribution of the supra-second layer to the multiscale temporal scaffolding process at stake in beat and meter perception.

As depicted in Fig. 1, the proposed scheme comprises a faster pulse layer (hereafter referred to as the *subdivision layer*) concerned with single time intervals delimited by the successive events making up the rhythmic input, which can serve as a basis to map fast periodic pulses at a sub-second timescale^4,16,17^. The intermediate pulse layer (hereafter called the *beat layer*) is concerned with the local arrangement of these successive events (also referred to as groups)^4^. These local arrangements can be considered as unsyncopated or syncopated (also referred to as strongly periodic or weakly periodic in the beat layer), depending on the extent to which these groups match the periodicity of the perceived beat^22,24^. Finally, the slower pulse layer (hereafter referred to as the *cycle layer*) is concerned with longer, seamlessly repeating, or looped, rhythmic patterns, which can serve as a basis for mapping slow periodic pulses at a supra-second timescale^4,15,25^. Notably, the boundaries between these three layers are flexible and depend on a multitude of factors pertaining to the stimulus (e.g., speed, timbre, etc.) and to the listener (e.g., cross-cultural or inter-individual differences, knowledge of the perceptual conventions inherent in musical genres, etc.). Nevertheless, there is generally a considerable alignment between the timescale of temporal cues in the rhythmic input and the timescale of perceived periodic pulses, particularly at the beat and subdivision layers^2,4,12^.

Thus far, most neuroimaging studies have focused on the role of temporal cues at the intermediate beat layer, while keeping periodic recurrence at the slower layer highly prominent through the use of looped rhythmic patterns^8,10,11,13,26,27^. However, little effort has been focused on evaluating the effect of supra-second temporal cues on the internal representation of beat. Yet, repetition of rhythmic patterns represents a core component of music^25,28–30^, one that is key to the aesthetic appeal and social power of music^5,31^. Repeating rhythmic patterns are often easily recognized and learned without requiring explicit training, and this periodic recurrence can help to structure musical pieces and anticipate future events^4,28,32^.

Therefore, we hypothesize that the slow periodicity provided by the repetition of rhythmic patterns serves as a supra-second temporal cue to the beat. This cue would offer a temporal anchor point onto which faster periodic pulses at the beat layer could be mapped by interpolation, through multiscale temporal scaffolding potentially involved in beat perception^4,33,34^ (see also expectancy-based^35,36^, or neural oscillations theories^7,23,37^). Note that the term “slow periodicities” is used here as a comprehensive term referring to the periodic recurrence in the acoustic signal arising from pattern repetition, rather than to specific mechanisms of beat perception.

We tested the role of these slow periodicities by recording behavioral and neural responses in healthy human adults. Behavioral measures of the beat representation were obtained by asking participants to tap their finger along with the most prominent periodic pulse they would perceive while listening to rhythmic sequences^7,38^. In a separate session, neural responses were captured using electroencephalography (EEG) while the same participants were listening to the same rhythmic sequences, from which neural representation of the perceived beat were measured using frequency analysis (see^12,39^ for reviews).

These behavioral and neural measures were compared across three sequences presented in separate conditions. In the three sequences, temporal cues to the beat layer were invariantly low (i.e., weakly periodic, syncopated rhythms), whereas the periodic recurrence in the slower cycle layer corresponding to rhythmic patterns was manipulated to gradually decrease across stimuli (sequence #1: repetition of a 4.8 s pattern; sequence #2: repetition of a 9.6 s pattern; and sequence #3: no pattern repetition). A decrease in the behavioral and neural measures across conditions would thus demonstrate a critical role of slow periodic cues in beat processing. Alternatively, observing comparable beat-related responses across conditions would suggest that periodic cues at the faster beat and/or subdivision layers may be sufficient to enable beat representation.

We also tested how these supra-second temporal cues interacted with other factors assumed to play a role in beat processing, namely, short-term prior context^40^ and long-term musical expertise. To this end, different presentation orders of the conditions were compared, thus allowing us to test whether supra-second temporal cues would be used as temporal anchors for the beat not only when these slow cues are actually present in the rhythmic input but also in subsequent sequences where these cues are relatively degraded (Fig. 3). Additionally, group comparisons across professional musicians and non-musicians aimed to test the extent to which long-term musical practice overrides the lack of slow temporal cues in the rhythmic input, allowing for the representation of a stable internal beat template irrespective of sensory input degradation^2,41^.

## Materials and methods

### Participants

To measure the long-term shaping of beat perception by music practice, two groups of 26 healthy volunteers recruited from the Brussels area (Belgium) participated in this study. The first group consisted of professional musicians (17 females, 5 left-handed, 27.54 ± 6.18 years old, 19.5 ± 5.46 years of musical practice, and 26.75 ± 7.8 h of musical practice per week—mean ± standard deviation) who were either studying or had graduated from a music conservatory (i.e., bachelor’s or master’s degree) at the time of the experiment. The second group consisted of non-musicians (17 females, 2 left-handed, 25.96 ± 5.53 years old, 0 ± 0 years of musical practice, and 0 ± 0 h of musical practice per week). Both groups were matched for dance experience (musicians: 2.34 ± 3.89 years of dance experience and 1.15 ± 2.96 h of weekly practice; non-musicians: 0.81 ± 2.00 years of dance experience and 0.60 ± 1.16 h of weekly practice; both *p*-values > 0.05). Participants had no history of hearing, neurological or psychiatric disorders, and provided written informed consent before the start of the experiment. This study was approved by the local ethics committee (“Comité d’Éthique Hospitalo-Facultaire Saint-Luc – UCL”, ref. 2018–353) and all experiments were performed in accordance with relevant guidelines and regulations.

### Auditory stimuli

**Fig. 2:**
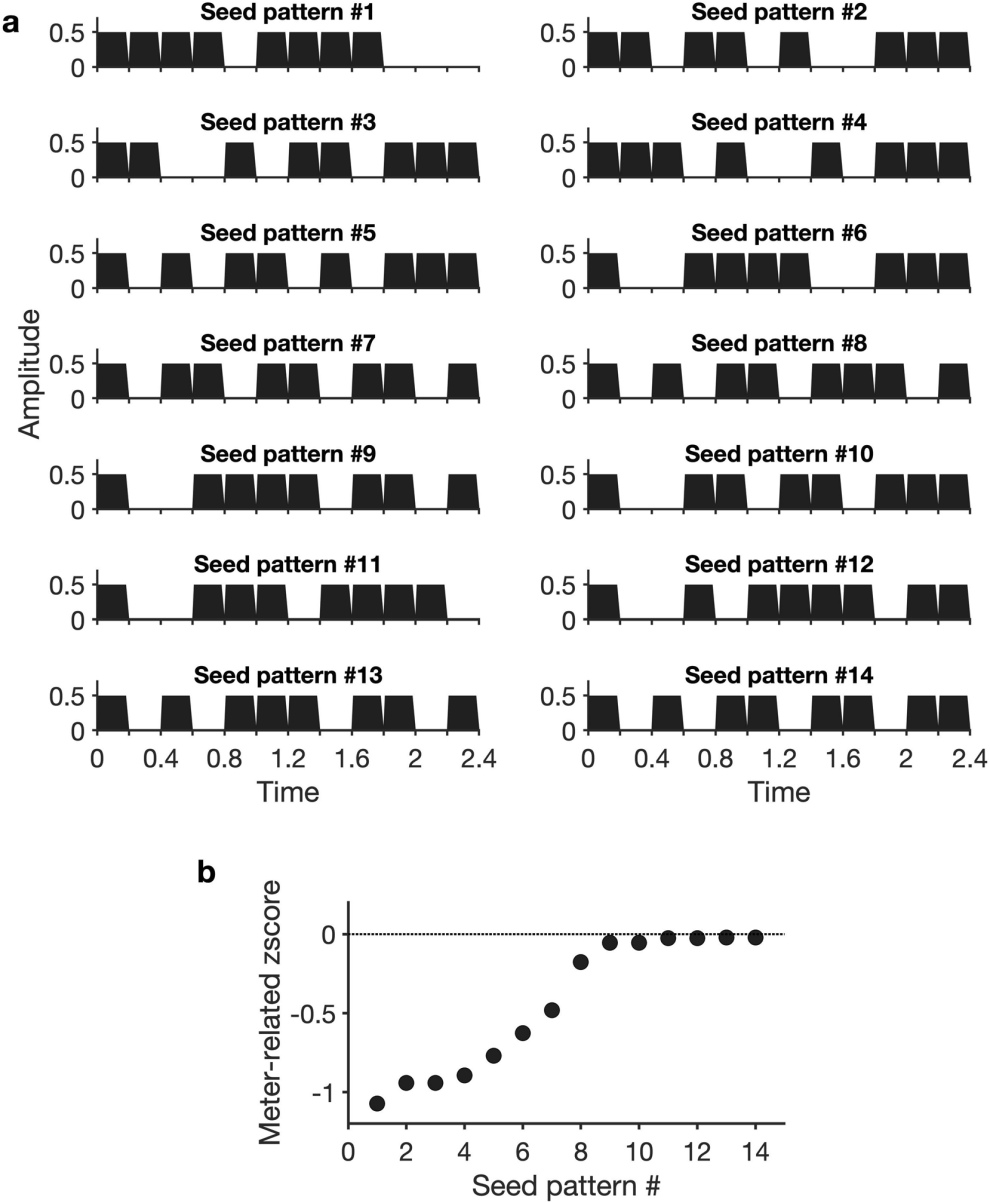
Temporal envelope of the fourteen seed rhythms. (**a**) All rhythms were based on a twelve-interval 200 ms grid structure (i.e., 2.4 s in total), with eight of the intervals consisting in sound events and the remaining four intervals consisting in silent events. The sound and silent intervals were arranged such as to yield syncopated rhythms. (**b**) Weak prominence of beat periodicities was confirmed through frequency analysis of the amplitude modulation of the rhythms, from which z-scores were calculated. All seed rhythms showed negative beat-related zscores, indicating weak prominence of beat-related periodicities in their modulation spectrum compared to beat-unrelated periodicities.

A set of 14 seed rhythms was created using Matlab (R2020a, *The MathWorks, Natick, USA*; Fig. 2; see electronic supplementary material 1 for details). Each rhythm was based on a 12-interval grid structure made of 200 ms evenly spaced time points and consisted of eight sound events, and four silent events. For each seed rhythm, the arrangement of sound and silent events on the 12-interval grid was manipulated to obtain 2.4 s syncopated (i.e., weakly periodic in the beat layer) rhythms.

Sound events consisted in a pure tone of 200 ms duration (10 ms and 20 ms linear ramps up and down). The carrier frequency of the pure tone varied between 150 and 200 Hz ([150.00, 161.19, 173.21, 186.12, 200] Hz) in each successive trial to increase participants’ vigilance due to the change in tone between trials. These relatively low tone frequencies were chosen since low tones have been shown to elicit brain responses with higher signal-to-noise ratios^42^, and stronger meter-related neural responses^8^. To account for the spectral sensitivity of the auditory system^43^, the intensity of each pure tone was equalized to 70 dB_A_ using a Bruel & Kjaer (type 2250, *Denmark*) sound level meter.

To confirm that temporal cues to beat-layer periodicities were weak in these stimuli (i.e., syncopation criterion), we measured the prominence of beat periodicities in the modulation spectrum of each rhythm. This was done by extracting the envelope of the acoustic signal of each 2.4 s rhythmic pattern using a Hilbert transform and subsequently applying a Fast Fourier Transform (FFT). The distribution of energy within the first 12 frequency bins (from the frequency corresponding to the duration of the rhythmic pattern, here 1/2.4 s = 0.41 Hz, to the 11th harmonics corresponding to the period of the grid timepoints, 1/0.2 s = 5 Hz) reflects periodic recurrences in the amplitude modulations of the acoustic signal^9,39,44,45^.

From this set of frequencies of interest, frequencies were classified as meter-related when corresponding to the most plausible metric pulses (1.25 Hz and harmonics, based on tapping behavior observed in studies using similar rhythms^8,10,13,46^), and as meter-unrelated frequencies otherwise. Amplitudes at all frequencies of interest were then converted to z-scores, and the z-scores at beat-related frequencies were averaged to serve as an index of their relative prominence in the modulation spectra (see^39^ for further details on this approach). All seed rhythms presented negative beat-related z-scores, indicating that the amplitude at beat-related frequencies was, on average, smaller than the average amplitude across all frequencies of interest. In other words, beat-related frequencies did not stand out relative to the whole set of frequencies characterizing the temporal structure of the rhythmic patterns. Additionally, this syncopation criterion was also corroborated by alternative syncopation measures (counterevidence C-scores; see electronic supplementary material 1 for details).

The 14 seed rhythms were then concatenated into three 67.2 s sequences that varied in terms of pattern repetition. The medium pattern repetition sequence consisted of a 4.8 s rhythmic pattern made of the concatenation of two of the seed rhythms (seed rhythms #5 and 10), which was seamlessly looped 14 times within the sequence. The long pattern repetition sequence comprised the same 4.8 s pattern from the medium pattern repetition sequence followed by two additional seed rhythms, thus forming a longer rhythmic pattern of 9.6 s (seed rhythms #5, 10, 8, 9), which was seamlessly looped 7 times within the sequence. Finally, the no pattern repetition sequence was made by randomly concatenating the 14 seed rhythms to generate the 67.2 s sequence (seed rhythms #6, 8, 11, 5, 12, 5, 7, 6, 11, 7, etc.).

All sequences were controlled (i) to yield similar beat-related z-scores, (ii) to not present identical rhythms following each other within a pattern, and (iii) so that the no-pattern repetition sequence would not reproduce parts of the pattern from the other two sequences. Note that the label “short pattern repetition” sequence was purposely avoided here, as it better refers to the duration of the seed rhythms (i.e., 2.4 s), which corresponds to the typical duration of rhythms used in previous similar neuroimaging studies^8,10,13,47^. Note also that a condition with repetition of such a 2.4 s rhythmic pattern was not included in the current study to maintain a reasonable experiment duration, and that longer patterns were hypothesized to more effectively target the 6–8 s limit for the implicit learning of rhythmic patterns^15,48^. Audio files of the stimuli are available on an online repository (https://osf.io/yz4hd/?view_only=52d6f29db48f4ccbb6dfdae6d5f8328e).

To test whether low-level sensory processing from early stages of the auditory pathway could explain the substantial enhancement of beat-related frequencies observed in neural responses compared to the input^13,39,49^, all three sequences were analyzed using a cochlear model. This model estimated the sound representation at the level of auditory nerve fibers (as implemented in the Auditory Toolbox for Matlab, with 128 characteristic frequencies distributed from 130 to 16,000 Hz^50^). The output of this model was averaged across the cochlear channels, and the signal was transformed into the frequency domain by applying an FFT. The beat-related z-score was then computed for each stimulus condition to confirm the lack of prominent beat-related frequencies, thus excluding possible low-level confounds in the EEG results.

### Experimental design

**Fig. 3:**
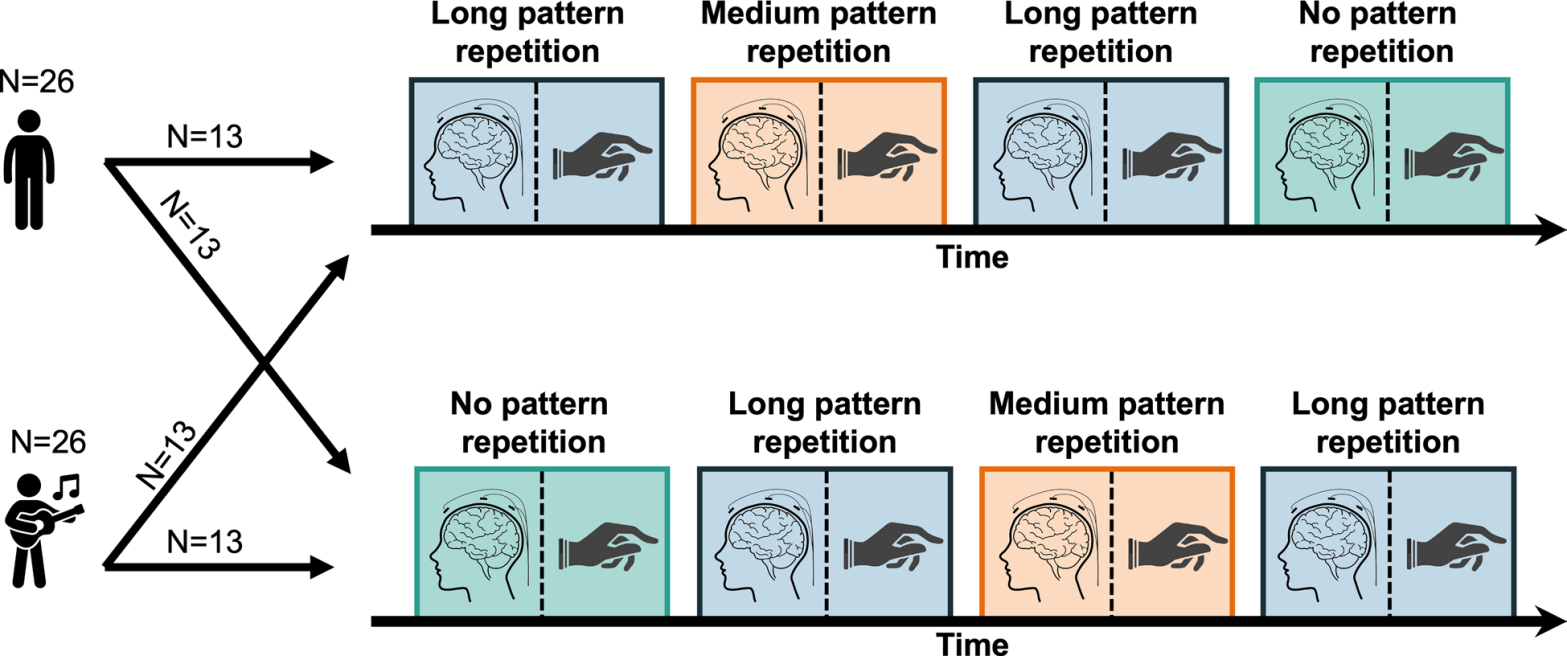
Experimental design. Musician and non-musician groups of participants were presented with four blocks in two possible fixed orders—the top and bottom condition orders providing maximum and minimum prior context, respectively, with the no pattern repetition condition positioned either at the end or the beginning of the block design. Half of each group was assigned to each condition order, and for each condition neural activity was recorded using electroencephalography, followed by a tapping session.

The experiment took place in a single session. Each participant listened to all three sequences presented in blocks, and the block corresponding to the long-pattern repetition condition was presented twice (Fig. 3) to test for effects of short-term context. Another way to test for possible effects of short-term context was to compare two different fixed orders of the blocks, with the no pattern repetition condition placed either at the beginning (i.e., no prior context) or at the end of the experiment (i.e., maximum prior context). Within each group of participants (musicians vs. non-musicians), half of each group was assigned to a different condition order, thus yielding a mixed factorial design.

Each of the four resulting blocks consisted of 10 EEG trials followed by 3 tapping trials. During EEG trials, participants were seated in a comfortable chair and were asked to listen carefully to the rhythmic sequences while fixating on a visual target placed in front of them and avoiding unnecessary head or body movements. To maintain participants’ attention to the temporal properties of the stimuli during EEG trials, participants were asked to report whether they heard a tempo change, and, if so, the direction of this change at the end of each trial^8,40,51^. These tempo changes were introduced in an additional sequence snippet appended to the end of 6 out of 10 trials (3 accelerations, 3 decelerations, and 4 trials without tempo changes). Modulations of the tempo were implemented following a 4.8 s long cosine waveform with a fixed magnitude for musicians (minimum and maximum grid intervals of 188.7 and 217.4 ms, respectively), and an adaptative two-down, one-up staircase method^52^ (grid intervals starting at 169.5 for accelerations and 238.1 ms for decelerations with adaptative steps of 8 ms) for non-musicians to account for their reduced ability to detect tempo modulations as compared with musicians^53^. Note that these additional snippets were excluded from further analyses, thus exclusively leaving responses to rhythmic inputs with a steady tempo for the analyses. Before the start of the experiment, participants were familiarized with several examples of these tempo changes applied to syncopated rhythms that were not used in the actual experiment.

For the tapping trials, participants were instructed to tap the regular pulse that was spontaneously perceived while listening to the stimulus by producing up and down movements with the index finger of their preferred hand on a custom-built response box (i.e., the exact instructions were: “*Tap the index finger of your preferred hand in time with the regular pulse that you perceive when listening to the rhythm, as if you were clapping your hands on the music during a live performance*”). The experimenter remained in the recording room with the participant at all times to monitor compliance with the procedure and instructions.

### Tapping recording and analysis

Tapping responses were recorded using a custom-built response box made of a hard tapping surface (i.e., producing auditory feedback, mitigated by the ear inserts used to deliver acoustic inputs simultaneously) and a pressure sensor underneath. From the tap time series, intertap intervals (ITIs) were calculated as durations between successive taps. For each trial, participant and condition, the median ITI was taken as an index of the perceived beat period^2^.

Additionally, we converted the tap time series to a sequence of phases relative to the participant’s predicted beat, determined by the meter periodicity closest to their median ITI. The circular mean was then taken as an index of tapping stability, with values ranging from 0 to 1, indicating low and high synchronization with the rhythmic input, respectively^7,54^.

#### EEG recording and preprocessing

The EEG was recorded using a Biosemi Active-Two system (Biosemi, Amsterdam, Netherlands) with 64 Ag–AgCl electrodes placed on the scalp according to the international 10/20 system, and two additional electrodes placed on the mastoids. EEG recordings were preprocessed using Matlab (R2020a, The MathWorks, Natick, USA) and Letswave (http://letswave.org).

First, signals were digitized at a sampling rate of 512 Hz. EEG recordings were high-pass filtered offline at 0.1 Hz (4th order Butterworth filter) to remove slow drifts, and segmented from 0 to 67.2 s relative to the trial onset (excluding any appended snippets with tempo changes relevant for the perceptual task). Signals were then re-referenced to the average of mastoid electrodes to increase the signal-to-noise ratio of neural responses to acoustic rhythms in frontocentral channels, which would later be selected^55,56^.

Excessively noisy channels were visually selected and linearly interpolated across all trials and conditions, separately for each participant (maximum 3 channels interpolated in 2 participants). Artifacts attributed to eye blinks and horizontal eye movements were identified and removed using independent component analysis^57^ based on visual inspection of their typical waveform shape and topographic distribution. For each participant and condition separately, the epochs were averaged across trials in the time-domain to reduce the contribution of background noise that was not time-locked to the stimuli, thus improving the signal-to-noise ratio of the EEG activity^58^.

The signal was then averaged across a set of 9 frontocentral channels (‘F1’, ’Fz’, ’F2’, ’FC1’, ’FCz’, ’FC2’, ’C1’, ’Cz’, ’C2’ in the 10–20 international system) based on prior observations of brain responses to rhythmic sound patterns being predominantly captured by these channels^8,10,38^. Finally, the averaged waveforms were transformed into the frequency domain using an FFT, and baseline corrected by subtracting the average amplitude at neighboring bins (2^nd^ to 5^th^) on both sides of each frequency bin, in order to locally correct for the background noise in the EEG signal^58^.

#### Relative amplitude of the beat-related neural activity in the frequency domain

A vast majority of previous EEG studies using a frequency-tagging approach to investigate the neural representation of beat have used repeated rhythmic patterns^8,10,11,13,26,27^. In such a context, the selection of frequencies of interest is relatively straightforward, because the response to a repeated rhythmic pattern should reliably recur at a fixed, known period corresponding to the pattern duration. This repeated response can be conveniently isolated in the frequency domain, as it only contains peaks at frequencies corresponding to the pattern repetition rate and harmonics.

Critically, the distribution of amplitudes across these frequencies of interest is related to the shape of the response elicited *within* each repetition of the rhythmic pattern. Moreover, the degree of periodic recurrence of the response (i.e., beat periodicities) within each pattern cycle is reflected in the relative prominence of amplitudes at a subset of frequencies of interest (i.e., beat -related frequencies), which correspond to the rate of this nested recurrence and its harmonics. To measure such prominence at the rate of the perceived beat periodicities (as directly informed by the group-averaged median ITI), the amplitudes across all frequencies of interest are typically standardized as z-scores and averaged across beat-related frequencies (i.e. frequencies corresponding to the rates and harmonics of the beat, see^43^ for the importance of analyzing harmonics in complex, non-sinusoidal signals). Notably, the resulting mean z-score quantifies the amplitude at beat-related frequencies *relative to* the amplitude at other, beat-unrelated frequencies, which are expected to emerge in the response spectrum due to stimulus repetition, but do not capture beat periodicities. The mean beat-related z-score thus constitutes a normalized index of the prominence of beat periodicities in the EEG signal^9,12,39,59^.

Along this line, we determined a set of beat-related frequencies based on the tapping, namely the observed convergent ITIs across conditions*.* However, determining beat-unrelated frequencies based on the harmonics of the pattern repetition rate would be ill-suited here, as each condition contained a different repetition rate (up to a complete absence of pattern repetition), thus precluding comparisons across conditions due to the profound differences in the number of beat-unrelated frequencies. Yet, beat-unrelated frequencies are key to standardizing response amplitudes at beat-related frequencies using a z-score, thus capturing the prominence of beat periodicities irrespective of unit and scale^39^. Therefore, guided by recent work^46,60^ emphasizing the importance of high signal-to-noise ratio for methods characterizing brain responses, we adopted a data-driven approach to selecting frequencies of interest and overcoming this imbalance without any prior assumption about possible beat-unrelated frequencies.

First, we identified the most prominent peaks from the group-averaged EEG spectrum of each condition. These prominent frequencies were obtained by running the *findpeak* function implemented in Matlab, with a minimum peak prominence set as the mean plus two standard deviations of the spectrum between 0 and 30 Hz. This measure was computed separately for each group of participants, and only frequencies present in both groups were retained. The same procedure was applied to the modulation spectrum of each of the three rhythmic sequences, and the series of prominent peaks from the three stimuli were merged with those extracted from their corresponding EEG spectra to better capture the input/output transformation. This way, small peaks at the stimulus level that are boosted in the EEG, or otherwise, prominent peaks in the input that are reduced in the output are both considered as frequencies of interest.

Furthermore, prominent peaks obtained below 1.25 Hz (i.e., pulse period corresponding to a grouping of 4 events, or 800 ms) were excluded from further analysis for several reasons. First, including the pattern repetition rates of 0.104 Hz and 0.208 Hz (i.e., the slow and medium pattern repetition conditions, respectively) as beat-related frequencies would have required including all their harmonics in the set of beat-related frequencies, thereby leaving no control (beat-unrelated) frequencies against which to further standardize the beat-related frequencies^39^. Second, the frequency of 0.625 Hz (i.e., a pulse period of 1600 ms, or grouping of 8 events) could not be clearly classified as either beat-related or unrelated, as no participant tapped at such a slow rate. Third, lower frequencies were expected to be particularly susceptible to 1/f noise in the EEG signal. In addition, peaks at 5 Hz and harmonics were also excluded as they are likely to be mostly driven by the shape of the average response to individual sound events making up the stimuli.

The steps described thus far could, in principle, yield a rather different number of prominent peaks (i.e., frequencies of interest) across conditions. To avoid such imbalance, we retained only the smallest number of frequencies of interest among conditions by (i) selecting those with the largest amplitude in the modulation spectrum of each stimulus, and (ii) forcing the inclusion of frequencies matching the median ITIs tapped by participants. From this set of prominent peaks, two frequencies corresponded to the convergent tapping beat rates (i.e., 1.25 and 2.5 Hz), and one (i.e., 3.75 Hz) corresponded to a harmonic of these rates, resulting in 3 beat-related frequencies for each condition. All remaining 24 frequencies were aggregated as beat-unrelated frequencies.

Finally, the amplitudes of all these frequencies of interest were converted into z-scores and averaged separately for the beat-related frequencies (frequencies corresponding to the period of the median ITIs, and prominent harmonics – 1.25, 2.5 and 3.75 Hz) and beat-unrelated frequencies (all non-beat-related frequencies of interest). As expected, this procedure yielded a different selection of beat-unrelated frequencies across conditions. However, the fact that this selection was conducted within an identical spectral range and was restricted to an identical number of these frequencies across conditions ensured fair comparison of the relative prominence of beat-related frequencies across conditions.

The frequency-tagging approach, as implemented here using magnitude spectrum analysis, has been shown to be sensitive to aspects that are unspecific to the relative prominence of periodicities, such as the shape of the response, thus potentially biasing the obtained results^39^. To overcome this specificity issue, a novel implementation of frequency-tagging based on autocorrelation has been developed recently, providing a measure of periodic recurrence that is invariant to the shape of the response. While this novel implementation provides greater specificity, it shows limited application in the case of non-repeating patterns (see^39^ for a detailed account). A magnitude spectrum analysis was therefore used in the present study, as a relevant approach to measure periodicity with high sensitivity, assess the stability of neural phase-locking, and enable direct comparisons between signals of different nature, unit and scale, which is key to a quantitative investigation of input–output transformations (see ^12,39^ for reviews on these methods).

Keeping the overall sequence duration constant across conditions necessarily resulted in a greater number of medium, as opposed to long patterns presented to the participant. To ensure that any differences across conditions could not be attributed to this exposure effect, supplementary analyses were conducted after equalizing the number of pattern presentations across conditions. Specifically, the same analysis was carried out after retaining only the first 5 (out of 10) trials from the medium-pattern repetition condition, thus ensuring a total of 70 patterns repetition in both the medium-pattern and long-pattern repetition conditions (14 patterns per trial × 5 trials in the medium-pattern repetition condition; 7 patterns per trial × 10 trials in the long-pattern repetition condition). The no-pattern repetition condition was excluded from this control analysis, as selecting only 10 repeated patterns (1 pattern per trial × 10 trials) would have resulted in an insufficient amount of data in the other conditions.

### Statistical analysis

Statistical analyses were performed using R (version 4.2.1) with the significance level set at p < 0.05. To account for the absence of normal distribution in the behavioral results, non-parametric Mann–Whitney tests were used for comparisons across the two groups, and between the two condition orders, and a non-parametric Friedman test was used for comparisons across conditions. For neural responses, a three-way mixed ANOVA was conducted to evaluate the main effect and interaction of the factors condition, musical expertise, and condition order. Where relevant, the Greenhouse–Geisser correction was used to correct for violations of sphericity in the performed ANOVAs. Post-hoc comparisons were carried out using paired t-tests, and a false discovery rate (FDR) correction was applied when needed to adjust for multiple comparisons.

## Results

### Behavioral results

**Fig. 4:**
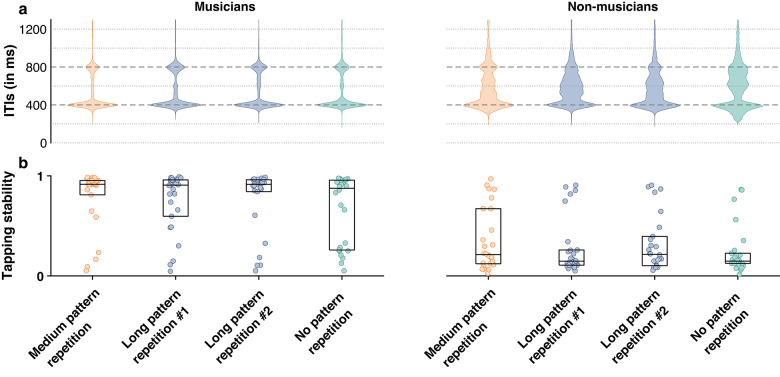
Behavioral results: non-musicians showed overall poorer stability at tapping the beat along with the rhythmic inputs, as compared to musicians. (**A**) Distribution of intertap intervals (ITIs) across participant groups and conditions. Horizontal lines correspond to integer multiples of the 200-ms grid structure on which rhythmic patterns were built, thus corresponding to plausible beat rates participants could have synchronized their tapping to. Dashed lines highlight 400- and 800-ms, corresponding to the convergent tapping rates across participants. (**B**) Tapping stability. Values ranging from 0 to 1 indicate low and high synchronization with a steady periodic beat, respectively. The horizontal line and limits of the boxplot indicate the median and 25th and 75th percentiles, respectively.

Figure 4A depicts the distribution of inter-tap intervals (ITIs) for both groups of participants across all four conditions. The observed convergent median ITIs at the group level and across conditions were thus used to inform the selection of beat-related frequencies for the frequency analysis of neural responses. Specifically, these ITIs corresponded to 1.25 Hz (i.e., 1/800 ms, or 4 times the grid interval) and its harmonics (thus also including 2.5 Hz, corresponding to 1/400 ms, or 2 times the grid interval), in line with previous studies^13,40,46,60^.

To evaluate behavioral responses, the tapping stability (i.e., the vector strength of the circular asynchrony between tapping onsets and the nearest hypothetical pulse position) was compared between groups, condition orders, and across conditions (Fig. 4B). Professional musicians showed higher stability than non-musicians in all conditions (Medium pattern repetition: W = 564, *p* = 1.442e-05, *η*^2^ = 0.574 ; Long pattern repetition #1: W = 578, *p* = 3.304e-06, *η*^2^ = 0.609; Long pattern repetition #2: W = 574, *p* = 5.111e-06, *η*^*2*^ = 0.599; No pattern repetition: W = 580, p = 2.644e-06, *η*^2^ = 0.614). However, within-group comparisons across conditions and condition orders showed no significant effect (Condition, Musicians: χ^2^(3) = 1.57, *p* = 0.667; Condition, Non-musicians: χ^2^(3) = 3, *p* = 0.39; Condition order: W = 5784, *p* = 0.387), although some non-musicians appeared to strongly benefit from the order offering maximum prior context, leading to a bimodal distribution of tapping asynchronies and a close to significant interaction (Order: Group: W = 1608, *p* = 0.09).

Together, these results suggest that the degradation of pattern repetition, as well as prior context, did not play a significant role in tapping performance. Only musical expertise had a positive effect, as observed by the more clustered ITIs and increased tapping stability measured in the musicians group.

### Frequency Analysis of the Stimulus and EEG responses

**Fig. 5:**
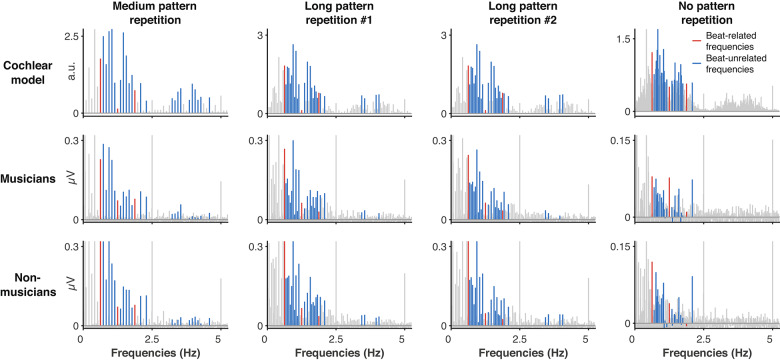
Modulation spectra obtained using cochlear model for each stimulus condition (top row), and their corresponding EEG responses in musicians (middle row), and non-musicians (bottom row). Frequencies of interest are respectively highlighted in red or blue for beat-related or beat-unrelated frequencies. EEG spectra correspond to a pool of 9 frontocentral channels and are averages over the 26 participants in each group.

**Fig. 6:**
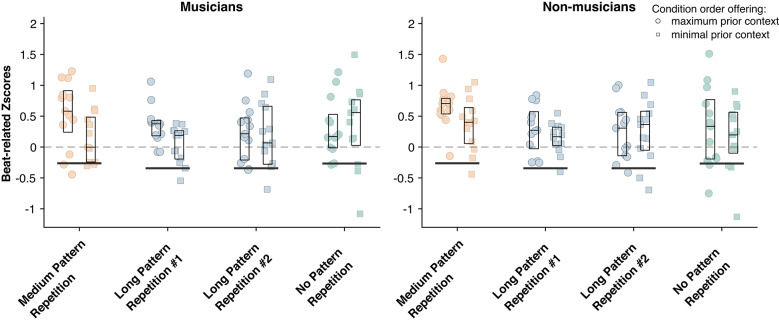
Mean z-score of EEG responses at beat-related frequencies across groups (left plot: musicians, right plot: non-musicians), for all conditions and condition order (circles: condition order maximizing prior context, squares: condition order minimizing prior context). Corresponding values obtained from the cochlear model output are shown as vertical black lines. The horizontal line and limits of the boxplot indicate the median and 25th and 75th percentiles, respectively. Beat-related z-scores above 0 indicate a larger prominence of beat-related frequencies than beat-unrelated frequencies, and vice versa.

Figure 5 displays the modulation spectra across stimulus conditions obtained from the cochlear model (top row) and EEG responses (musicians and non-musicians in the middle and bottom rows respectively). As expected, the degradation of pattern recurrence causes the acoustic energy (and the corresponding output of the cochlear model) to be spread across a larger number of frequency bins that is directly determined by the duration of the repeated pattern in the sequence (Fig. 5, top row), hence the necessity for a proper adjustment of a method for selecting frequencies of interest as described in the materials and methods section. After running the findpeak function on the modulation spectrum of each stimulus condition and their corresponding group-averaged EEG spectra, the medium pattern repetition condition showed the fewest prominent peaks (n = 27), which determined the number of frequencies of interest that would then be selected in each condition. After classifying 1.25 Hz and harmonics as beat-related frequencies (Fig. 5 in red, n = 3, as informed by tapping results), and all other frequencies of interest as beat-unrelated (Fig. 5 in blue, n = 24), the modulation spectrum of the cochlear model responses showed a weaker prominence of beat-related frequencies compared to beat-unrelated frequencies. This observation was confirmed by the negative beat-related z-score obtained in all four conditions (Fig. 6, horizontal black line), indicating that the rhythmic sequences contained little cues to the beat layers tapped by most participants.

One-sample one-tailed t-tests were then performed to estimate whether beat-related frequencies were more prominent than predicted by the cochlear model, which could not be explained by lower-level tracking of prominent acoustic features of the stimuli^12^ (Fig. 6, black horizontal line). Both groups showed a selective enhancement of beat-related frequencies in the EEG in all four conditions and for both condition orders (all p-values < 0.05, FDR corrected, Table 1). These results align with previous work showing a periodization of the input, even when the input shows weak beat periodicities in its modulation spectrum^9,11,12^. However, these results go one step further by showing that the processes underlying periodization persist in response to stimuli with degraded periodicities at the slower cycle layer, where periodic recurrences associated with pattern repetitions typically occur.

**Table 1:** Selective enhancement of EEG beat-related zscores observed in each group, condition and condition order.

		Mean	SEM	T-statistic	*p*-value (FDR corrected)	*p*-value control analysis (FDR corrected)
Musicians						
Medium pattern repetition	Maximum prior context	0.798	0.150	5.324	2.621e−04***	7.459e−06***
	No prior context	0.423	0.112	3.765	1.797e−03**	5.176e−02
Long pattern repetition #1	Maximum prior context	0.698	0.086	8.149	1.244e−05***	4.048e−03**
	No prior context	0.380	0.083	4.607	6.036e−04***	2.930e−03**
Long pattern repetition #2	Maximum prior context	0.574	0.128	4.501	6.452e−04***	3.406e−04***
	No prior context	0.521	0.146	3.559	2.420e−03***	4.360e−03**
No pattern repetition	Maximum prior context	0.575	0.130	4.425	6.628e−04***	
	No prior context	0.636	0.185	3.435	2.822e−03**	
Non-musicians						
Medium pattern repetition	Maximum prior context	0.923	0.096	9.651	4.197e−06***	1.002e−05***
	No prior context	0.631	0.120	5.274	2.621e−04***	4.360e−03**
Long pattern repetition #1	Maximum prior context	0.638	0.107	5.965	1.313e−04***	4.360e−03**
	No prior context	0.485	0.069	7.032	3.658e−05***	4.360e−03**
Long pattern repetition #2	Maximum prior context	0.591	0.126	4.675	6.036e−04***	1.462e−03**
	No prior context	0.599	0.140	4.275	7.846e−04***	7.804 e−03**
No pattern repetition	Maximum prior context	0.576	0.179	3.225	3.887e−03***	
	No prior context	0.411	0.150	2.738	8.994e−03***	

The *p*-value and *p*-value control analysis refer to the selective enhancement of the main analysis pipeline and the control analysis correcting for the total number of pattern repetition across conditions, respectively. The *p*-value is flagged with one star (*) if lower than 0.05, two stars (**) if lower than 0.01, and three stars (***) if lower than 0.001. SEM = Standard Error of the Mean.

Finally, we estimated the effect of these slow temporal recurrences, along with effects of musical expertise and prior context, on the amplitude of EEG responses at beat-related frequencies (Fig. 6). The three-way mixed ANOVA with the factors condition, group, and condition order showed a main effect of condition (F(3,48) = 2.711, p = 0.0470, *η*^*2*^*p* = 0.053), with larger beat-related z-scores observed in the medium-pattern repetition compared to the first presentation of the long-pattern repetition condition (t(51) = 3.411, *p* = 0.006, *η*^2^ = 0.472, FDR corrected), as well as a main effect of condition order (F(1,48) = 4.828, *p* = 0.033, *η*^*2*^_*p*_ = 0.088), with greater enhancement of beat-related frequencies in the order that provided maximum prior context (i.e. starting with the medium pattern repetition condition; t(103) = 2.553, p = 0.006, *η*^2^ = 0.250). However, no main effect or interaction was found for the group factor, suggesting that listeners’ neural responses may be similar irrespective of levels of musical expertise.

To ensure that these effects were not merely driven by differences in the total number of pattern repetitions across conditions, we conducted a supplementary analysis controlling for exposure effects. One-sample one-tailed t-tests showed similar selective enhancement of beat-related frequencies in the EEG than what was predicted by the cochlear model (Table 1, control analysis column). The three-way mixed ANOVA then yielded a main effect of condition (F (2,48) = 2.711, *p* = 0.0470, *η*^2^_*p*_ = 0.07), with larger beat-related z-scores in the medium-pattern repetition condition compared to the first presentation of the long-pattern repetition condition (t(51) = 2.482, *p* = 0.049, *η*^2^ = 0.344, FDR corrected). Similarly, the main effect of condition order was also significant (F(1,48) = 4.607, *p* = 0.037, *η*^2^_*p*_ = 0.088), with higher beat-related z-scores observed in the order providing maximum prior exposure to supra-second periodic structure (t(77) = 2.332, *p* = 0.022, *η*^2^ = 0.264). These supplementary analyses thus confirm that the effects observed in the initial analysis remain robust even when controlling for differences in the total number of pattern presentations. In other words, these results cannot be solely explained by a greater exposure to the medium-pattern repetition condition but rather corroborate the effect of pattern repetition on the neural beat representation.

While the data-driven selection of frequencies of interest adopted here is supported by recent methodological work^46,60^, alternative selections are possible. In the original analysis, the 3rd harmonic of 1.25 Hz (i.e., 3.75 Hz) was included because it emerged from the stimulus and/or EEG spectra, whereas the 3rd harmonic of 2.5 Hz (i.e., 7.5 Hz) did not. As requested during the review process, we repeated the analysis while including both 3rd harmonics (3.75 and 7.5 Hz) in the set of beat-related frequencies. This additional analysis yielded very similar results, confirming the robustness of our findings with respect to frequency selection (see electronic supplementary material 2 for details).

## Discussion

The current study investigated the role of rhythmic pattern repetition in beat processing. A selective enhancement of the neural activity at beat-related frequencies was found in all conditions, with pattern repetition further reinforcing the neural representation of the beat. Moreover, we observed stronger neural emphasis of the beat-related periodicities when the highly repetitive stimuli were presented first, indicating a carry-over effect from the directly preceding condition, which offered maximal prior short-term context in terms of pattern repetition^40^.

### Periodization of rhythmic input in neural activity

Results revealed that the relative prominence of beat-related frequencies was enhanced in neural activity as compared to their relative prominence in the input. Notably, this enhancement was observed in all conditions, irrespective of the degree of pattern repetition in the input. Crucially, while active debate remains on the computational mechanisms underlying the observed neural transformations of the rhythmic inputs, these neural responses cannot be explained by mere tracking of prominent periodicities of the rhythmic input, as all stimuli were purposely designed to lack prominent temporal cues at the intermediate beat layer (i.e., syncopated rhythms). This periodization of the rhythmic input observed in neural activity as captured with EEG thus arguably reflects processes related to beat perception, beyond sole lower-level sensory tracking of prominent features of the input^13,39^.

Importantly, the selective enhancement of beat-related frequencies was observed even in the non-repeating condition, which only provided a low degree of periodic recurrence in the input. In other words, it appears that pattern repetition is not necessarily required for listeners to periodize rhythmic inputs. Instead, periodic recurrence, even when limited only to faster layers, can already be sufficient to elicit significant neural emphasis of the beat. Together, these findings thus reveal processes through which the brain would leverage periodic recurrence restricted to a narrow timescale in the rhythmic input, to map pulse layers covering wider timescales^7,19,20,61^. This multiscale temporal scaffolding would thus play a critical role in the neural periodization of rhythmic input that could be experienced as the beat^9^.

To some extent, these findings may be related to the phenomenon of subjective meter (or “tick tock” effect), which refers to perception of periodic accents in unaccented isochronous rhythms^19,20,62^. More specifically, it could be hypothesized that, in the no-pattern repetition condition, interpolation of the prominent periodic recurrences at the fastest inter-onset-interval layer served as a basis to map an internal representation of a periodic structure at slower rates, thus yielding a set of nested periodic pulses. Such predictions are supported by models of neural resonance which posit that rhythmic inputs nonlinearly interact with intrinsic oscillatory networks, driving neural oscillations not only at the frequencies present in the stimulus, but also at integer ratio frequencies (i.e., integer multiples and divisors of the input frequencies)^7,11^.

Previous studies^63,64^ on Western populations have shown a clear tendency toward tapping rates corresponding to groupings of 2, 4, and 8 notes (compared with groupings of 3, 5, 6, and 7 notes). Corroborating these observations, the current behavioral results showed a convergence towards duple beats. However, the current study provides a nuanced differentiation from previous findings. Specifically, we found convergent tapping across participants at a rate corresponding to two times the fastest inter-onset intervals (Fig. 4A). This tapping rate is thus overall faster than that reported in previous studies which mostly found tapping rates corresponding to four times these intervals in response to repeated rhythms at the same tempo^8,10^. The faster rate observed here thus likely reflects an attraction toward the most prominent periodic recurrence available in the sensory input, here located at the fastest layer.

### Discrepancy between neural and behavioral responses

The behavioral measures revealed less stable sensorimotor synchronization performance in non-musicians than in musicians, which is consistent with previous research^2^. This cross-group difference in the ability to tap a periodic beat could appear contradictory with the neural measures which did not show such a contrast between the two groups. Rather, the neural measures showed “periodization” of the input that was significantly influenced by pattern repetition and prior context but not musical expertise.

Yet, these discrepancies between behavioral and neural measures align with the view of a default automatic periodization of the rhythmic input in neural activity, which would contribute to beat perception irrespective of musical expertise. Corroborating this view, previous research using intracerebral recordings in humans has provided evidence for such a periodization of rhythmic inputs at the earliest cortical stage of auditory processing^27^ (i.e., Heschl’s gyrus). These findings are also consistent with previous research showing a periodization of syncopated rhythms in the EEG of human adults even when the attention is focused away from the rhythmic input^49^, as well as in the EEG of 5–6 month-old human infants, despite the relative immaturity of higher-level associative and sensorimotor cortices at this developmental stage^47^.

Taken together, these studies suggest that the capacity to move to a periodic beat does not only rely on a default periodized neural representation of the rhythmic input as produced in the primary auditory cortex. Rather, this ability would also require strong functional coupling between auditory and higher-level associative and sensorimotor cortices, allowing this periodized representation to guide movement timing, thus in line with the functional definition of beat and meter in music^65,66^. The critical role of such functional coupling between these brain regions is supported by modelling work^7^, and corroborated by the evidence that body movement can shape the internal beat representation^6,51,67^. Despite similar neural responses between groups, lifelong music practice likely strengthens this functional coupling, enabling musicians to better translate their neural representation of meter into body movement^68^. On the contrary, the relatively degraded periodic structure at the intermediate and slow layers used in the current study as compared to previous studies^8,10,49^ may have proven too limited at enabling consistent motor entrainment to the beat in non-musicians.

In addition to the cross-group difference discussed above, we also found a cross-task difference. The positive effect of pattern repetition on the prominence of meter periodicities was found in the neural but not tapping responses. This discrepancy may stem from differences in task demands and affordance, as participants were instructed and allowed to move to the beat only during the behavioral measurements. Self-entrainment to the beat through tapping along may thus reinforce the listener’s beat representation, especially in the context of syncopated, and non-repeated patterns, possibly masking the effect of repetition on the tapping performance^69^.

### Pattern repetition in music: capitalizing on implicit learning to foster beat perception

The current study shows that repetition of rhythmic patterns facilitates the internal beat representation. Several influential theories in the rhythm perception field could account for this phenomenon. According to neural resonance theories, this facilitation could stem from the capacity of the brain to subdivide the slow, supra-second periodicities provided by repetition of the rhythmic patterns into faster pulses through nonlinear coupling with the stimulus^7,20,23,37^. On the other hand, expectancy-based theories propose that such facilitation could arise from anticipatory attending mechanisms, whereby prediction about forthcoming events would strengthen the internal representation of the beat^35,36^. Yet, this effect of pattern repetition is relatively small. This relatively small effect size could be explained by the fact that temporal cues at slow cycle layers, as used here, may have been already close to the limit beyond which periodic recurrence no longer facilitates beat perception^4^ (but see^70^ for extremely long timescale beat perception based on explicit theorization and training).

However, the human brain has proven to be remarkably proficient at recognizing and learning recurring patterns over time^44,71,72^. This phenomenon, referred to as implicit learning already occurs on a short timescale^72^, and does not require any specific training (see^73^ for a review of implicit learning in the context of music). Supporting this, effects of implicit learning were observed with auditory stimuli lasting up to 8.4 seconds^48^, thus comprising the duration of the medium pattern repetition condition (i.e. 4.8 s) used in the current study. The current results thus open to future research comparing neural and behavioral responses across a finer-grained range of durations of pattern repetition. For example, starting with approximately 2 s long patterns (as in^8,10,27,47,49^) could further clarify the role of implicit learning processes in fostering beat perception.

## Conclusion

Despite being a core component of music worldwide^25,30–32^, the repetition of musical patterns (melodic phrases, rhythmic figures, etc.) has often been overlooked in studies of rhythm processing. The current experiment aims to fill this gap by showing that, while the repetition of rhythmic patterns is not required for the forming of a neural representation of beat, it can strengthen this neural representation.

Importantly, and beyond implications in music contexts, our results reveal the capacity of the human brain to leverage slow periodicities from the rhythmic input at the supra-second scale, to map internal representations of faster periodicities at sub-second timescales. By highlighting the multiscale nature of temporal processes involved in rhythm processing, this study advances our understanding of high-level perceptual processes allowing groups of individuals to coordinate interaction and communication in a multitude of fundamentally human joint actions ranging from music and dance performance to ritual, work, and play.

## Supplementary Information

Below is the link to the electronic supplementary material.

Supplementary Material 1

## Data Availability

Preprocessed and analyzed data are available on a public OSF repository **(**https://osf.io/yz4hd/?view_only=52d6f29db48f4ccbb6dfdae6d5f8328e). Additionally, anonymized raw data is available upon request from Emmanuel Coulon (emmanuel.coulon@uclouvain.be). The scripts used to conduct the analyses and figures are available on a public GitHub repository (https://github.com/Manu-RnB/pattern_repetition.git).
