# Supplementary material for: Neural representation of the musical beat is facilitated but not contingent on the repetition of rhythmic patterns
Source: Sci Rep. 2025 Dec 17;16:1222. doi: 10.1038/s41598-025-30780-1 (PMC12789069; doi:10.1038/s41598-025-30780-1)
Supplement: Supplementary file 1 — Supplementary Material 1 [file 41598_2025_30780_MOESM1_ESM.docx]

**Electronic Supplementary Material for**

**Neural representation of the musical beat is facilitated but not contingent on the repetition of rhythmic patterns**

Emmanuel Coulon^1*^, Sacha Baum^1^, Tomas Lenc^1,2^, Rainer Polak^3^ and Sylvie Nozaradan^1,4^

^1^Institute of Neuroscience (IoNS), Université Catholique de Louvain (UCLouvain),

Brussels, Belgium

^2^Basque Center on Cognition, Brain and Language, Donostia-San Sebastián, Spain

^3^RITMO Centre for Interdisciplinary Studies in Rhythm, Time and Motion, University of Oslo, Oslo, Norway

^4^International Laboratory for Brain, Music and Sound Research (BRAMS), Montreal, QC, Canada

**Supplementary Material 1 – Seed rhythm generation**

To generate the pool of seed rhythms, we first created in Matlab all possible 12-interval grid structures of sound and silent events (coded as 1 and 0, respectively), with the first interval always fixed as a sound event. This resulted in 2,048 candidate rhythms.

We applied a series of constraints to this set. We retained only rhythms containing eight sound events and four silent events, and excluded rhythms containing five or more consecutive sounds and three or more consecutive silences. To avoid redundancy, we further removed patterns that were phase-shifted versions of one another.

This procedure yielded 36 distinct rhythms. From these, we selected the 14 rhythms with negative beat-related z-scores according to the frequency-domain method described in the manuscript.

These frequency-domain measures were complemented by another measure that has been used in a number of studies to assess the complexity of rhythmic patterns, in particular the syncopation criterion^[1-4]^. This measure, called counterevidence score or C-score^[1]^, does not rely on the continuous time series of the stimulus but rather on a representation of the rhythmic patterns that is abstracted from the actual acoustic features composing the continuous stimulus time series, to further quantify the degree of mismatch between a putative metrical interpretation and the arrangement of sound events in the rhythmic pattern grid. In other words, this measure calculates the extent to which the grid points corresponding to sound onsets do not match with the putative beat. This measure thus yields higher C-scores for rhythms that are considered as more complex or syncopated. To provide a reference value for comparison, we added to the table below the C-score obtained from a simple, unsyncopated rhythm of the same grid structure as in the seed rhythms (xxx.xxx.xx.., where “x” and “.” represent sound and silent events, respectively)^[5-7]^.

| Stimulus | C-score | |
| --- | --- | --- |
|  | Beat rate of 1.25Hz | Beat rate of 2.5Hz |
| Medium Pattern Repetition | 126 | 280 |
| Long Pattern Repetition | 91 | 280 |
| No Pattern Repetition | 98 | 259 |
| Reference Unsyncopated Rhythm | 28 | 140 |

**Supplementary Material 2 – Alternative selection of frequencies of interest**

For transparency, we provide here an additional analysis requested by a reviewer, who suggested harmonizing the selection of harmonics across beat-related frequencies. Specifically, because a peak was detected at 3.75 Hz (i.e., the 3rd harmonic of the converging clapping ITIs at 1.25 Hz) in the stimulus and/or EEG spectra, the reviewer asked us to replicate our analyses while also including 7.5 Hz (i.e., the 3rd harmonic of the other converging clapping ITIs at 2.5 Hz), in order to maintain symmetry in the harmonics selection. However, including frequencies which do not significantly stand out from noise in either the stimulus or the EEG spectra, such as 7.5 Hz here, risks adding noise to the analysis rather than capturing the periodicity of the response.

When repeating the analysis with 7.5 Hz included in the beat-related frequencies, all groups, conditions, and condition orders still showed a significant selective enhancement of beat-related neural activity compared to the stimulus (Table 1 of the supplementary analysis).

Additionally, the repeated-measures ANOVA yields very similar results, with a main effect of condition (F(3,48) = 3.269, p = 0.023, η^2^𝑝 = 0.064) and larger beat-related z-scores observed in the medium-pattern repetition compared to the first presentation of the long-pattern repetition condition (t(51) = 3.828, p = 3.54e-5, d = 0.530, FDR corrected), and an marginally significant main effect of the condition order (F(1,48) = 3.959, p = 0.052, η^2^𝑝 = 0.076). Apart from this slight increase in the p-value, just above 0.05, for the condition order, all other results remained unchanged by the inclusion of 7.5 Hz.

7.5 Hz was not included in the original analysis because it did not significantly emerge from the noise. As expected, adding this frequency that primarily reflects noise rather than a periodic signal slightly weakened the observed effects, but the main results and their interpretation remain unchanged.

Given that this is the first study to assess neural responses to non-repeating rhythmic patterns, we consider a data-driven approach the most appropriate for capturing the input/output transformation and in line with recent EEG frequency-tagging methodological papers^[8,9]^.

| **Musicians** |  | **T-statistic** | **p-value**  **(FDR corrected)** |
| --- | --- | --- | --- |
| Medium pattern repetition | Maximum prior context | 6.178 | 2.37e-05*** |
|  | No prior context | 4 .272 | 5.42e-05*** |
| Long pattern repetition #1 | Maximum prior context | 10.380 | 1.20e-07*** |
|  | No prior context | 5.811 | 4.16e-05*** |
| Long pattern repetition #2 | Maximum prior context | 5.109 | 1.29e-04*** |
|  | No prior context | 5.031 | 1.47e-04*** |
| No pattern repetition | Maximum prior context | 7.886 | 2.18e-06*** |
|  | No prior context | 5.071 | 1.37e-04** |
| **Non-musicians** |  |  |  |
| Medium pattern repetition | Maximum prior context | 10.055 | 1.69e-07*** |
|  | No prior context | 6.001 | 3.10e-05*** |
| Long pattern repetition #1 | Maximum prior context | 6.855 | 8.80e-06*** |
|  | No prior context | 10.678 | 8.78e-08*** |
| Long pattern repetition #2 | Maximum prior context | 5.917 | 3.53e-05*** |
|  | No prior context | 6.311 | 1.94e-05*** |
| No pattern repetition | Maximum prior context | 5.392 | 8.11e-05*** |
|  | No prior context | 6.028 | 2.98e-05*** |

Table 1: Selective enhancement of EEG beat-related zscores observed in each group, condition and condition order for the supplementary analysis requested by Reviewer #1which includes 7.5Hz in the set of beat-related frequencies. The p-value is flagged with one star (*) if lower than 0.05, two stars (**) if lower than 0.01, and three stars (***) if lower than 0.001

**References**

1. Povel, D.J., Essens, P. Perception of Temporal Patterns. *Music Perception: An Interdisciplinary Journal* **2**, 411–440 (1985).
2. McAuley, J.D., Semple, P. The effect of tempo and musical experience on perceived complexity. *Australian Journal of Psychology* **51**, 176-187 (1999).
3. Matthews, T.E., Witek, M.A.G., Heggli, O. A., Penhune, V. B., & Vuust, P. The sensation of groove is affected by the interaction of syncopation and audio features. *PloS one***14**, (2019).
4. Hoddinott, J.D., Grahn J.A. Neural representations of beat and rhythm in motor and auditory cortices. *Cerebral Cortex* **34**, (2024).
5. Nozaradan, S., Schwartze, M., Obermeier, C., & Kotz, S. A. Specific contributions of basal ganglia and cerebellum to the neural tracking of rhythm. *Cortex* **95**, 156-168 (2017).
6. Lenc, T., Keller, P. E., Varlet, M. & Nozaradan, S. Neural tracking of the musical beat is enhanced by low-frequency sounds. *Proc Natl Acad Sci* **115**, 8221–8226 (2018).
7. Lenc T, Peter V, Hooper C, Keller PE, Burnham D, Nozaradan S. Infants show enhanced neural responses to musical meter frequencies beyond low-level features. *Developmental Science* **26** (2023).
8. Zhou, H., Melloni, L., Poeppel, D., & Ding, N. Interpretations of frequency domain analyses of neural entrainment: Periodicity, fundamental frequency and harmonics. *Front. Hum. Neurosci.* **10**, (2016).
9. Retter, T. L., Rossion, B., Schiltz, C. Harmonic amplitude summation for frequency-tagging analysis. *Journal of Cognitive Neuroscience* **33**, 2372- 2393 (2021).
